# Preparation and characterization analysis of biofuel derived through seed extracts of Ricinus communis (castor oil plant)

**DOI:** 10.1038/s41598-022-14403-7

**Published:** 2022-06-30

**Authors:** Saka Abel, Leta Tesfaye Jule, Lamessa Gudata, Nagaprasad Nagaraj, R. Shanmugam, L. Priyanka Dwarampudi, B. Stalin, Krishnaraj Ramaswamy

**Affiliations:** 1 Department of Physics, College of Natural and Computational Science, Dambi Dollo University, Dembi Dolo, Ethiopia; 2 Centre for Excellence-Indigenous Knowledge, Innovative Technology Transfer and Entrepreneurship, Dambi Dollo University, Dembi Dolo, Ethiopia; 3 Department of Mechanical Engineering, ULTRA College of Engineering and Technology, Madurai, Tamil Nadu 625 104 India; 4 TIFAC, CORE-HD, Department of Pharmacognosy, JSS College of Pharmacy, JSS Academy of Higher Education and Research, Nilgiris, Ooty, Tamil Nadu India; 5 Department of Pharmacognosy, JSS College of Pharmacy, JSS Academy of Higher Education and Research, Nilgiris, Ooty, Tamil Nadu India; 6 Department of Mechanical Engineering, Anna University, Regional Campus Madurai, Madurai, Tamil Nadu 625 019 India; 7 Department of Mechanical Engineering, Dambi Dollo University, Dembi Dolo, Ethiopia

**Keywords:** Biochemistry, Biological techniques, Biotechnology, Energy science and technology, Engineering

## Abstract

The current study assesses the prospect of using R. Communis seed oil as a substitute fuel for diesel engines. Biodiesel is prepared from the R. Communis plant seed oil by a single-step base catalytic transesterification procedure. The investigation deals with the Physico-chemical characteristics of R. Communis biodiesel and has been associated with the base diesel. It has been perceived that the characteristics of biodiesel are well-matched with the base diesel under the ASTM D6751 limits correspondingly. R. Communis biodiesel is blended in different proportions with base diesel such as D10, D20, D30, D40, D50 and D100 and is tested in a Kirloskar TV1 single-cylinder, 4 blows DI engine under altered loading conditions. Outcomes demonstrate that BTE and BSFC for D10 as well as D20 are similar to base diesel. BSFC indicates that the precise BSFC of base diesel, D10, D20, D30, D40 and D50 was 0.87, 1.70, 2.60, 3.0, 3.4, and 3.5 kg/kW-hr, respectively. The extreme BTE at full load condition for base diesel, D10, D20, D30, D40, D50 and D100 are 28.2%, 28.1%, 27.9%, 25.5%, 24.1%, and 23.6% , respectively. In the case of engine emissions, R. Communis biodiesel blends provided an average decrease in hydrocarbon (HC), Carbon-monoxide (CO) and carbon dioxide (CO2) associated with base diesel. Nevertheless, R. Communis biodiesel blends discharged high stages of nitrogen oxide (NOx) compares to base diesel. Base diesel, D10, D20, D30, D40, D50, and D100 had UBHC emissions of 45 ppm, 40 ppm, 44 ppm, 46 ppm, 41 ppm, and 43 ppm, respectively. The reduction in CO emissions for D10, D20, D30, D40, D50 and D100 are 0.13%, 0.14%, 0.17%, 0.18% and 0.21% respectively. The dissimilarity in NOx attentiveness within brake powers for D10, D20, D30, D40, and D50 and base diesel are 50-ppm, 100 ppm, 150 ppm, 250 ppm, 350 ppm, and 500 ppm, respectively. The dissimilarity of CO_2_ emanation with reverence to break powers for the base-diesel, D10, D20, D30, D40, D50, and D100 are 4.8%, 4.9%, 4.8%, 4.56%, 4.9% and 5.1%, respectively. The present research provides a way for renewable petrol blends to substitute diesel for powering diesel engines in that way dropping the reliance on fossil fuels.

## Introduction

In the recent decade, urbanization, modernization and industrialization linked to energy production and utilization have been a fundamental loop in various economic, scientific and social sectors^1^. The reduction of nonrenewable fuel bases, attended with greenhouse gas emanations, has a dangerous issue^2,3^. Thus, the essential change for discovering substitute opportunities to overawe the world scale pending energy disaster, allowing for the environmental distresses and its qualification, while challenging the strengthening energy mandate has an imperative requirement of the time^4^. Consistent, inexpensive, harmless, and ecofriendly energy deliveries are important for the financial growth of a population and for the global welfare of an individual^5^. A foremost part of the global electricity mandate is being encountered with the aid of using fossil fuels such as coal, fossil gasoline, and other petroleum outputs. Compression-Ignition (C.I.) engines powered with the aid of using petrodiesel are usually hired in industry, agriculture, and transportation subdivisions because of their flexibility in members of the family of better gasoline productivity, steadfastness, cost-effective, and harmless procedures. Scientists are inspired to research the potential of engines significantly to solve environmental problems. Moreover, biodiesels are a hopeful substitute fuel source that encounters manufacturing necessities, particularly tailpipes emission, contributing to as well as influencing weather changes and distinctive pollution. Also, biodiesels are considered carbon–neutral fuels since the emission linked with their ignition are comparatively the same as the CO_2_ emission absorbed during the growth of the plants from which the biodiesels is prepared. Furthermore, the quick usage of fossil grounded fuel sources in the current time has been initiated to raise the atmosphere's dangerous emissions, mostly in the form of CO_2_, thus escalating the greenhouse effect and global warming^6,7^. Henceforth, it is crucial to carry out an extra fuel whose powerfulness be constructed from possessions nearby worldwide among the nation like alcohol, biodiesel and vegetal oils^8^. Biodiesel delivers a realistic yield power as well as expressively decreases engine radiations contrast to other diesel. Hence, biodiesel composites have been developed as substitute gasoline to decrease releases in diesel engines. Biodiesel can be organized from natural oil, animal oils or fats, tallow, and leftover cookery oil. The procedure takes vicinity to differentiate those oils from biodiesel or biofuel and is a concept of transesterification^9^. Biodiesel has many environmentally beneficial properties^10^; it includes in one of the major bases of energy assets in the biosphere as it about provisions 14% of the biosphere’s energy depletion^11–13^. Numerous studies are being given for endorsing biofuels. Biofuels, biodiesel has extra energy, as it has belongings identical to the conduct of diesel gasoline^14^. Biodiesel is biodegradable, renewable and more eco-friendly than petroleum grounded fuel^15^. There are more than 350 oil-bearing produces that have been known, among which only Pongamia, jatropha curcas, Neem, Hemp, Mahua, Calophyllum inophyllum and cottonseed oils are taken as a potential alternative fuel for diesel engines^16^. Apart from the renewability, the benefits of biofuels are as follows: High oxygen content, higher flash points and higher lubricity that yield complete combustions in contrast with conventional diesel fuels^17^. Moreover, the environmental benefits are another study influence because of the lesser greenhouse effect, less air pollution, less contamination of water, and soil and reduced health risk^18^. According to literature, it is instigated that the majority of the research is carried out in the form of the various petroleum in diesel devices, particularly biodiesel constructed from diverse varieties of natural oils, and truly constant paintings have been accomplished on biodiesel made from Cannabis sativa seeds^19^. Manufacturing hemp seeds into methyl esters were performed, and the physicochemical residences of hemp biodiesel were associated with other base diesel as well as pleasant biodiesel was determined to be located similar to ASTM D675^20^. Methanolysis of hemp seed oil catalyzed with the aid of using hydroxide was studied with the assistance of using the full fundamental compound rotatable strategy^21^. More favourable reaction temperatures and methanol to hemp seed oil molar ratio (43.4 °C and 6.4:1) than the latter (56.8 °C and 8.5:1) at somewhat higher catalyst loading (1.2% as opposed to 1.0%). R. Communis, in other names it is identified as castor bean) goes to the Euphorbiaceae species. It can be cultivated in numerous areas like; the USA, India, China, Central Africa, Brazil and Australia, through various growing systems^22^. Matured R. Communis seed is toxic to human health and animals because it contains toxic protein^23^. The R. Communis seeds crop is around 902 kg/ha yearly and its seed has about 47–56% oil. The emollient (oil) is viscid, somewhat odorous, light yellowish, non-volatile and non-drying, with an insipid taste^24^. Few reports have been suggested for the seed of R. communis biodiesel^25^.

**Figure 1:**
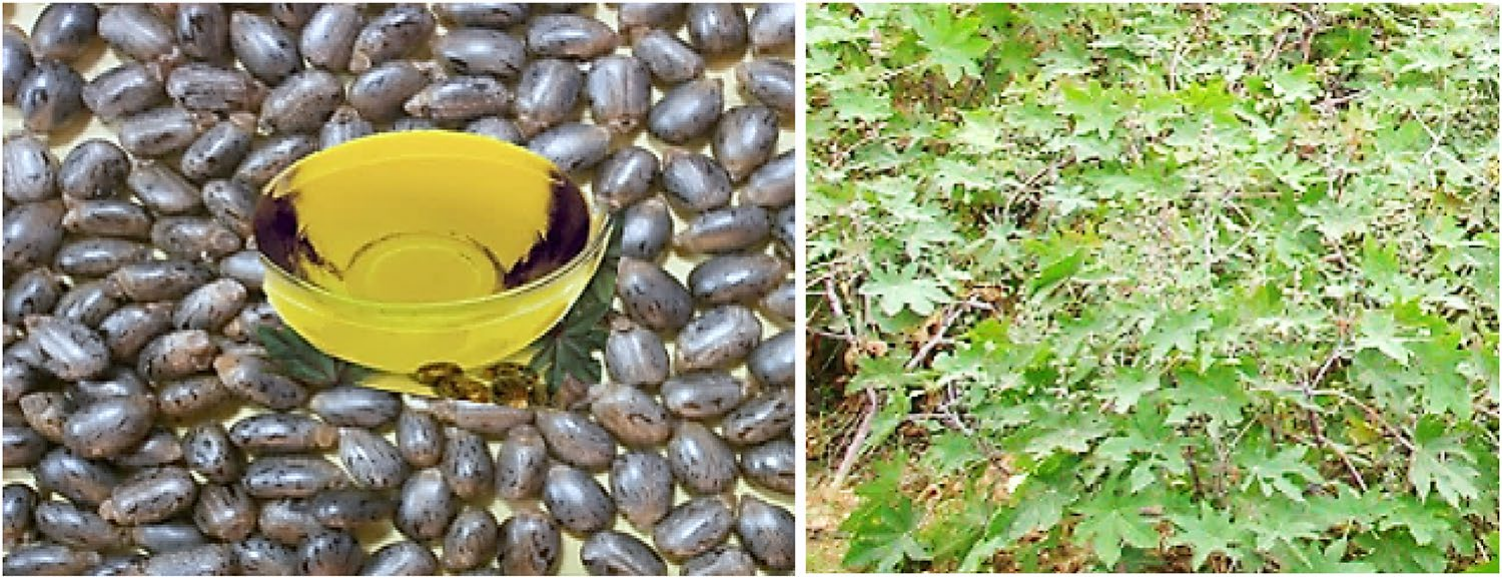
R. Communis Seed has taken from Oromia Region, East Wollega Zone Gudaya Bila, Ethiopia.

This plant originates in Africa; however, it is located in each wild and cultivated state altogether, the tropical and subtropical nations of the planet. In wild situations, this plant is well-tailored to arid conditions and is able to face lengthy durations of drought. R. communis plants can gift precocious, median, and not on time cycles^26^. The precocious cycle is that inside which flowering happens approximately forty-five days after sowing. However, in Ethiopia, it has no suggestion, as it can be the source of biofuel in a simple method. The median cycle affords flowers at an intermediate time among the precocious and not on a time cycle, which allows a flowering time of ninety to one hundred twenty days after sowing. A current study specializes in biodiesel of the seed of R. communis^27,28^. The goal of this research is to evaluate whether it is feasible to use organic waste from R. communis seed oil as a substitute energy source for internal combustion engines in the near future. Employing renewable petroleum combinations to exchange diesel for the purpose of powering diesel engines, as evidenced by this research, can reduce or eliminate our dependence on fossil fuels. R. communis Seed taken from the Oromia Region, East Wollega Zone Gudaya Bila Woreda, and Ethiopia is illustrated in Fig. 1. Around this area, nobody had done research on R. Communis biodiesel. The selected tree is simply used for other purposes. The researchers were inspired to prepare biodiesel from R. Communis. Categorizing bases of biofuels such as biodiesel and biochar can possibly decrease the environmentally friendly influences of fossil fuels^29^. Biological fuels can hostage the accumulative use of fossil possessions and avoid heaviness on nonrenewable bases^30,31^. Though it is imperative to use applied, scientific as well as vigorous apparatuses to assess the actual benefits of biofuels over conservative energy bases, Life cycle assessment (LCA) has been selected as a wide-ranging evaluation method to calibrate ecological influences over the whole manufacture sequence of bio-fuels^32^. Economically, biomass ignition is not the finest policy to use biomass due to causing plain environmental pollution and not retrieval of the total energy warehoused in the substrates^33^. Hence, this research aims to censoriously assess present biomass to biofuel paths as well as associated studies which evaluated environmental impacts for the whole cycle. Life cycle assessment tools for bio-waste operation methods were studied in different countries^34^. Carbon-based waste, municipal solid waste, chicken meat, and Biomass waste, was done in Germany, Italy, Serbia, Catalonia and London used as feedstock respectively^35^, and Economic analysis respectively. This results in a life sequence portfolio motorized; Organic conduct, Waste converts all torrents into important possessions^36^. This was effective manipulation of agro-waste residual biomass; Low cost and clean bioethanol manufacture. The results reported with the advantage of approaches were in comparison altered wastes organization ways; Sympathy examination to assess the effect of possible enhancements; documentation of criticalities as well as development possible, Classify and enumerate the ecological influences^37^. Certain ecological influence capacities are intended like global-warming probable, ozone cover exhaustion and increasing energy mandate. The ecological performance of the different bio-waste treatment machinery is encompassed as a choice quantity in bio-waste administration predicting, energy effective departure methods for the enterprise of ecofriendly, chemical procedures application of maintainable feed-stocks with extraordinary alteration reactions as well as energy basis for the maintainable production of bio-chemical^38^.

The main objectives of this study were to study the manufacture and analysis of biofuel derived through seed extracts of R. communis**,** using diesel blend on the emanation features and ignition enactment of a cylinders C.I. engine under several circumstances. Additionally, an engine that consume emissions like nitrogen oxides (NOx), carbon monoxide (CO), hydrocarbons (HCs) and carbon dioxide (CO_2_) was analyzed. The influence of usage of clean diesel, as well as diesel blends with methyl esters on engine appearances such as brake thermal efficiency(BTE), brake specific fuel consumption(BSFC) as well as engine power, was, examines with present LCA investigations and high-light current serious outcomes.

## Materials and methods

Materials required were, 300 g R. communis seed, 5 g of KOH, 10L Methanol, 1Pcs Magnetic stirrer, 10Pcs Beakers (different sizes), 20 L water, 0.15 g Nanoparticles, tin foil four Inch, 6Pcs Conical flasks, 3Pcs Stand funnel separators and Ultrasonicator. The plant we used in this study was cultivated in Ethiopia. This study obeys applicable official strategies, national global procedures, as well as regulations.

### Sample preparation

R. communis seeds gathered from the East Wollega Zone, Gudaya Bila Woreda, Darbes Kebele, Ethiopia. After being gathered, it truly became saved at ordinary temperature to dry. The dried out seed had been crashed, and the inner seed took out the peel, had been removed, with the aid of mortar and pistil, it truly became grinded to induce powder of the seed. R. Communis seeds are crumpled in an automated expeller tool to induce primary oil from the seeds. Biodiesel is synthesized from R. Communis oil with the aid of using the character step base-catalyzed transesterification technique. The solution of primary oil, 500 ml of methanol with the aid of using the ratio (4:1) and 5 g of KOH as the reagent was heated in an incredibly 1000 ml plump backside flask on an intellectual heater mixed with a magnetic-stirrer revolving at 700 rms with 1 min. The internal solution was enthused very well and warmed at 75 °C for a time length of 3 h. The solution became relocated to a centrifuge funnel, allowing glycerol to be remarkable with the aid of using severity for 3 h. State departure occurs with pinnacle layer biodiesel and bottommost layer related to waste glycerol and unreacted methanol, which might be strained out of the flask.

The biodiesel attained in the procedure is advance cleaned by deionized water for 8–10 turns to remove acids till the pH of water is reached and again the product is annealed above 150 °C to isolate the wetness of the biodiesel. Thus R. communis L. biofuel is gained.

### Test technique

**Figure 2:**
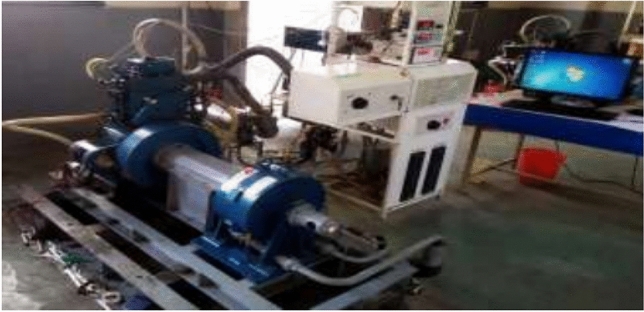
Illustrative interpretation of D.I Engine Arrangement.

The biofuels applied in the present research comprise diesel oil, and biodiesel besides their mixtures. An experimentations were conducted by well-ordered diesel as base fuel represented as Base Diesel, (15% biodiesel + 85% base diesel) signified as D10, (20% biodiesel + 80 base diesel) signified as D20, (30% biodiesel + 70 base diesel) signified as D30, (50% biodiesel + 50% base diesel) signified as D50, and (100% biodiesel) signified as D100 altered engine rated power. Two (2) fuels containers are used as storage of diesel petroleum as well as biodiesel individually with a burette and 3 ways stop elevate as expressed in Fig. 2 the prepared fuel is transferred from base-diesel to biodiesel by functioning separate regulators provided in each reservoir as well as three (3) way stop-clock. Beforehand consecutively the engine to the prepared bio-fuel, it was permitted to run for adequate time to consume the residual fragment of fuel since the aforementioned experimentation.

Laboratories were performed at detonation timings of 23 °C bTDC (before-Top-Dead-Centre) and inoculation stress of 300 bars. Then, equal examinations were accomplished with biodiesel and its mixtures. For each examination fuels and individual loads about three(3) times analyses were done to obtain an average value. While the engine touches the alleviated occupied disorder, routine constraints such as brake thermals efficiency (BTE), brake unique fuels consumptions (BSFC), exhaust gas temperatures (EGT) and emissions for HCs (hydro-carbons), CO_2_ (carbon dioxide), CO (carbon monoxide) and NOx (Nitrogen-oxide) were measured.

### Analysis of oil

The excellence of oil is expressed in terms of the Physico-chemical assets like as kinematic- viscidness, thickness; fattening value as well as flash-point of R. communis biodiesel were determined by following A.S.T.M (American-society-for-testing-materials) methods and compared with base diesel. It is realized that the Physico-chemical characteristics of biodiesel vary from conservative diesel; that can affect diesel engines piece as well as emanation physiognomies deprived of any extra adjustment requisite to work on the engines.

**Figure 3:**
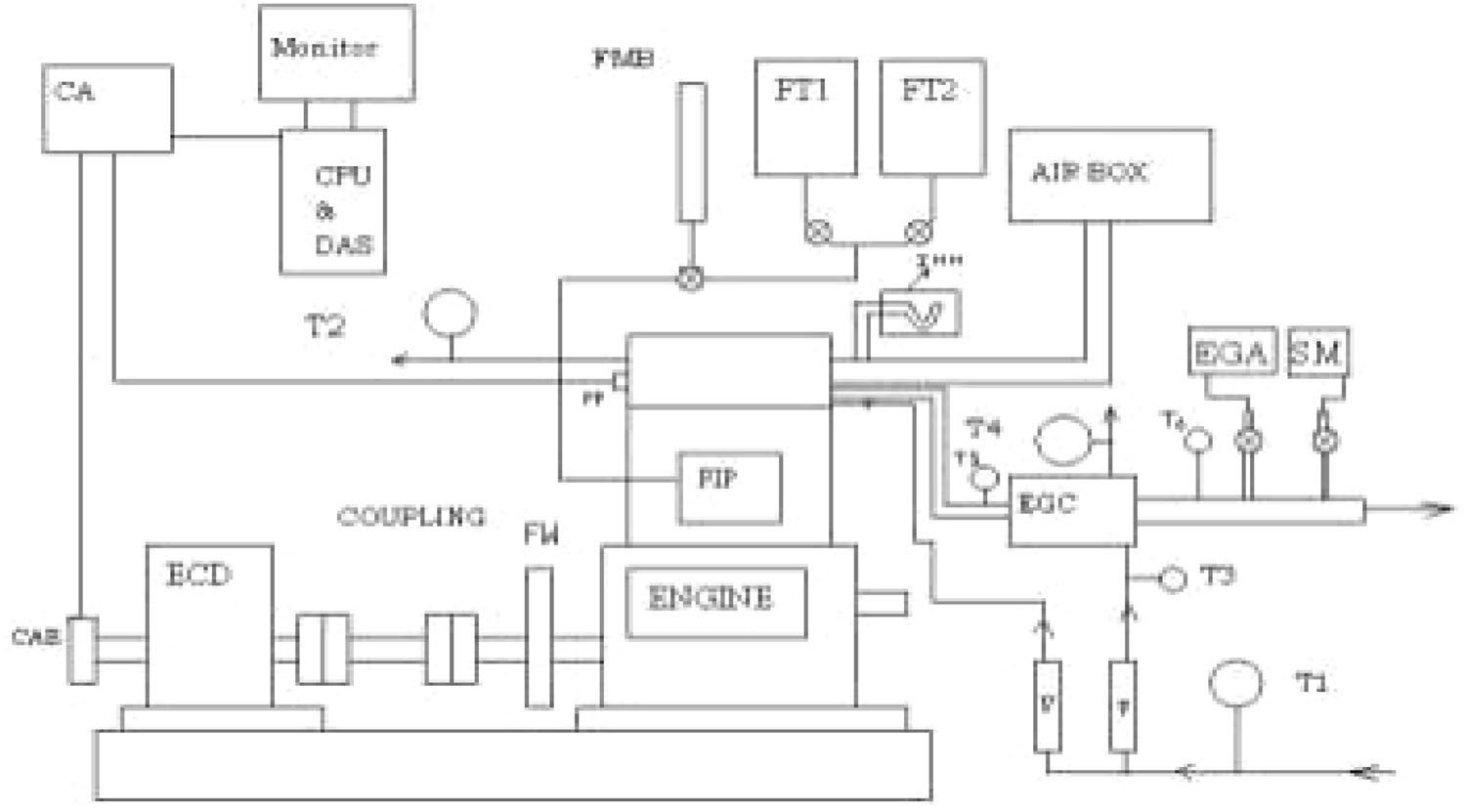
Schematic arrangement of D.I Engine setup.

The investigation is conducted experimentally on a normal Kirloskar TV1 single-cylinder with 4 strokes of regular pace diesel. Figure 2 shows the pictorial view of the DI engine setup. The engine is combined with a water air-conditioned whirlpool current dynamo-meter laterally with loads cells. With the assistance of the controller provided at the dynamometer, the load applied to the engine can be varied. A diagram representation of the laboratory procedure showed in Fig. 3. Exhaust fuel line emanations likes as HCs (hydro-carbons), CO_2_ (carbon dioxide), CO (carbon monoxide) and NOx (Nitrogen-oxide) were restrained through an AIR REX HG-540 gas emission analyzer. Engine enactment constraints such as BTE, BSFC, and EGT are Emissions are studied.

## Results and discussion

### Oil yield

The base oil received from the R. communis seeds and the use of a computerized expeller was deliberated to 45% oil earnings. After the trans-esterification technique, 90% of methyl ester yield was obtained with a molar proportion of methanol-oil 4:1. The essential instruments and explanations were displayed in Table 1. The following equation is used to calculate the yield of oil extraction.

$$ {\text{Yield }} = \, \left( {\text{Weight of refined biodiesel}} \right)/\left( {\text{weight of oil used}} \right) \, \times {\text{ a hundred Performance}} $$

**Table 1:** Test engine specification.

Essentials	Explanation
Engine-Model	Kirloskar TV1
Engine category	Single cylinder, Four stroke, DI engine
Windbag diameter	87.61 mm
Blow size	121.11 mm
Joining bar dimension	234.00 mm
Density proportion	18:1
Brushed capacity	661.45 cc
Graded-power	3.49 kW
Graded speediness	1499 rpm
Dynamo-meter	Eddy current
Oil ignition scheduling	23 °C bTDC
Oil inoculation pressure	200 bar

### Performance

#### Brake thermal efficiency (%)

**Figure 4:**
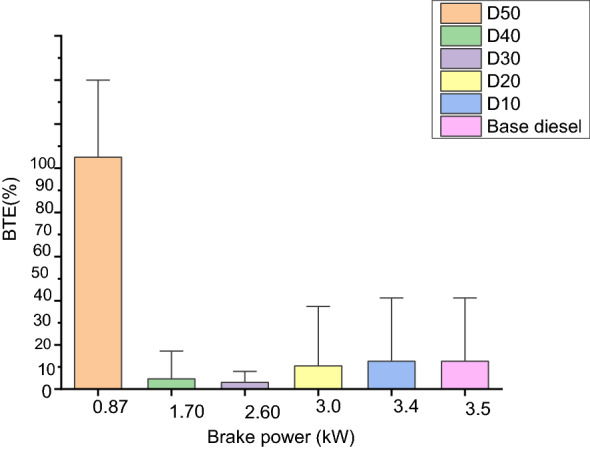
BTE vs. brake power.

Figure 4 shows the BTE with brake power current enactment is the ratio among the ability output and consequently, the electricity brought via the mechanical system, the final of the products of the inoculated fuel mass value and the decreased combustion value^39–42^.

It is perceived that BTE initially declines for every test fuel with the rise in loads that goes 80% as well as thereafter has a habit of rising with rising in loads. The highest BTE for the base diesel are achieved at full load in the following circumstances: D10, D20, D30, D40 and D50 are 28.2%, 28.1%, 27.9%, 25.5%, 24.1%, and 23.6%, respectively. It is clear from the graph that the BTE of base diesel is superior to R. communis methyl-ester blends when compared to each other. The reason that R. communis methyl-ester displays low performance is an excessive density, viscosity, and minor heating value of base-diesel. The greater viscidness finally ends up in reduced atomized, fuel evaporation, as well as ignition, and therefore, the BTE of R. communis methyl-ester is much less than that of base diesel^43^.

It is also placed in the vicinity of the base diesel, where the blends D10 and D20 are the closest. Therefore the change in BTE within base diesel, D10 and D20 blends is important at supreme loads. Though, the BTE of blended fuel is greater than that of well-ordered biodiesel. D40 is lower than all blends.

#### Brake specific fuels consumptions (kg/kWh)

**Figure 5:**
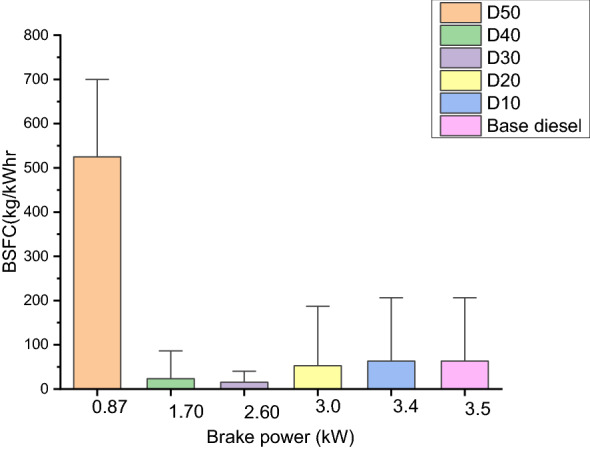
BSFC vs. brake Power.

The brake specific fuels consumptions (BSFC) the tangible form of fuels expended to yield 1 kW powering in 1 h^44^. Figure 5 indicates the distinction between base diesel and each biodiesel blend. It indicates that the precise BSFC of base diesel, D10, D20, D30, D40 and D50 was 0.87, 1.70, 2.60, 3.0, 3.4, and 3.5 kg/kWh, respectively. It is distinguished that, the BSFC of R. communis of each and every blend is greater than that of base diesel in different loading situations. However, EGT for D10 is greater than base-diesel because of greater viscosity.

The portion of R. communis methyl ester in the composite affects the engine's low value with improved enactment. Furthermore, this may be because of sufficient time for the whole burning of biodiesel at little engine rapidity and extended after sweltering level with higher viscidness^45^.

#### Exhaust gas temperature (EGT) (°C)

**Figure 6:**
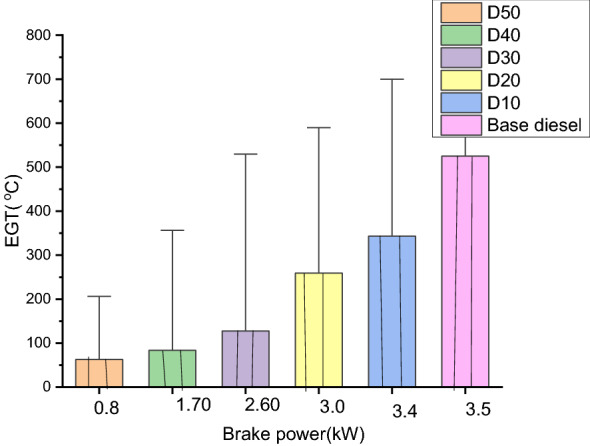
Exhaust gas temperature vs. Brake power.

The distinction of consuming fuel line hotness with esteem to break energy in line with time for sordid diesel, biofuel, and its blend is expressed in Fig. 6. The consumed biofuel temperature increases with the successful weight for all verified blends.

The Exhaust gas temperature (EGT) of D10 is greater than base diesel because of its greater viscosity, which results in minor atomization, minor evaporations and prolonged ignition. D10 confirmations 500 °C of temperature had been, as base biodiesel indicates 550 °C. R. communis methyl ester concentration is increased for D20, D30, D40, and D50 with increasing viscosity of the EGT. Furthermore, this is because adequate times for complete ignition of biodiesel at little engine rapidity as well as extended after ignition level with sophisticated viscidness^46^.

### Emissions

#### Unburned hydrocarbon emission (ppm)

**Figure 7:**
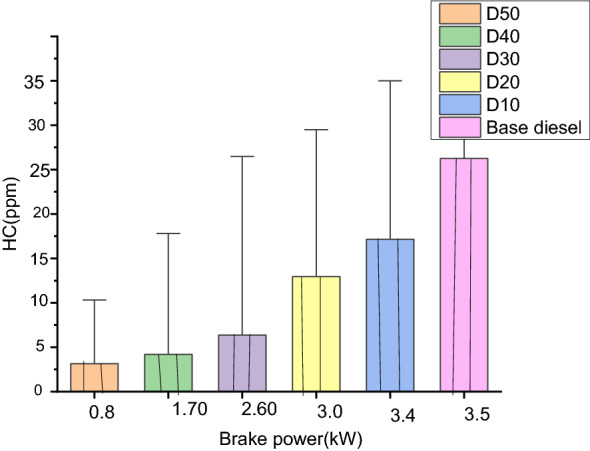
Relationship of unburned hydrocarbon (UBHCs) emissions for different blends.

As shown in Fig. 7, the percentages of unburned-hydrocarbon (UBHC) releases since base diesel merely in regard of R. communis blend are minor than those from diesel–fuel. Emissions of UBHCs are initiated to decline in partial engine loads scenarios as well as to rise in greater engine load situations^47^.

Base diesel, D10, D20, D30, D40 and D50 had UBHC emissions of 45 ppm, 40 ppm, 44 ppm, 46 ppm, 41 ppm, and 43 ppm, respectively. Base diesel's maximum HC emission at full load was 45 ppm, whereas D10's was 40 ppm. There was a 10% reduction in HC emissions, indicating that the biodiesel blend for D10 burns more efficiently. This may be due to there being oxygen comfortable in the biodiesel that causes speedy the ignition chemical reaction whole burning of the fuel^48^.

#### Carbon monoxide (%)

**Figure 8:**
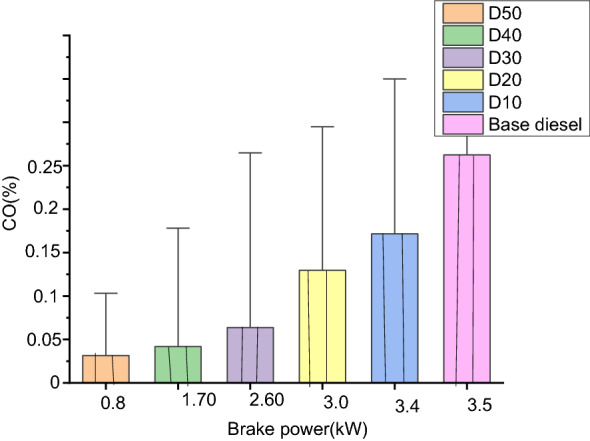
Comparison of carbon-monoxide emission for diverse blends of oxides of nitrogen (ppm).

Figure 8 displays the variance in carbon-monoxide (CO) of every investigated oil with regard to brake powers. The deviation in carbon-monoxide emanations for each and every biodiesel blend is equitably small; when compared with base diesel. As the loads are enlarged on the engines, there is intensification in carbon-monoxide emission for every test fuel. The rise in carbon-monoxide emissions level at greater loads is because of a gorgeous combination as lower loads resulted in partial ignition fuels. This is because of the existence of greater oxygen comfortable in biodiesel. The decrease in carbon-monoxide releases for D10, D20, D30, D40 and D50 are 0.13%, 0.14%, 0.17%, 0.18% and 0.21%, respectively. The minor carbon-monoxide emanations have been perceived within blended biodiesel fuels as well as the smallest in D10 and D20 blends, this may be because of oxygen comfortable and fewer Carbons to Hydrogen proportions of biodiesel that cases complete ignition. Nevertheless, it is shown that the declining tendency of carbon-monoxide release doesn’t depend on biodiesel measurement in combinations^49^.

#### Oxides of nitrogen (ppm)

NOx emanation is a general word for nitric-oxide (NO) and nitrogen oxide (NO_2_), which is formed from the rejoinder of nitrogen with oxygen gas airborne through a burning procedure. The supreme ignited gas temperatures, the comparative attentiveness of oxygen (O_2_) and rejoinder time are serious variables for oxide of nitrogen (NOx) creation.

**Figure 9:**
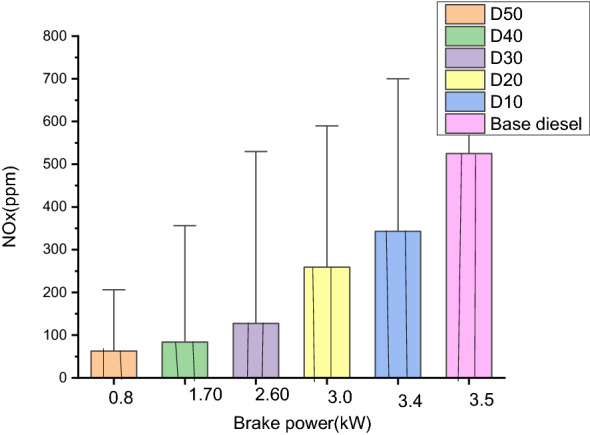
Comparison of the oxide of nitrogen emissions of various blends.

The dissimilarity in the oxide of nitrogen (NOx) attentiveness with brake-power for D10, D20, D30, D40, and D50 and base diesel is 50 ppm, 100 ppm, 150 ppm, 250 ppm, 350 ppm, and 500 ppm, respectively. As shown in Fig. 9. Oxide of nitrogen (NOx) emanations from every biodiesel blend is greater than that of conservative fuels. It has been discovered that the NOx emanation of D10 is greater than before by 2% in comparison to diesel at the esteemed loads and that the oxide of nitrogen (NOx) emanation of D10 is increased by 20% as a result of the proportion of biodiesel in blend^50^. A developed cetane amount would outcome in a compressed combustion interruption period thus permitting a smaller amount of time for fuel intercourse before the pre-mixed ignition stage. Therefore, feebler mixtures would be produced and burnt throughout the pre-mixed ignition stage causing comparatively abridged oxide of nitrogen (NOx) creation. It has been discovered that the existence of more oxygen (O_2_) within the molecule of biodiesel blends causes a rise in the oxide of nitrogen (NOx) releases^51^.

#### Carbon dioxide (%)

**Figure 10:**
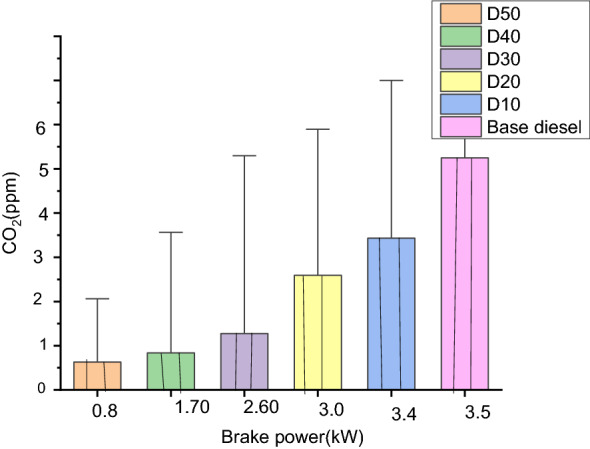
Comparison of Carbon dioxide emissions for various blends.

The influence of the biodiesel blends on Carbon dioxide (CO_2_) emanation is displayed in Fig. 10. Carbon dioxide (CO_2_) emanations were a smaller amount up to 30% loads after that, it was greater for D100 at extreme loads with a value of 5.1% by capacity. The variance of Carbon dioxide (CO_2_) emanation with reverence to brake-power for base-diesel, D10, D20, D30, D40, and D50 are 4.8%, 4.9%, 4.8%, 4.56%, 4.9% and 5.1%, respectively. The result obtained may be due to the existence of extra oxygen (O_2_) for the complete ignition of fuels and emanations growing progressively as the loads rise for each and every blend of fuel. Again this can be caused by the influence of poorer working temperatures with extraordinary latent heat evaporation. Carbon dioxide (CO_2_) is the significant constraint that specifies the ignition effectiveness of specific fuels. Greater Carbon dioxide (CO_2_) emission denotes improved combustions^52^.

From Table 2 the results basically show good agreement with previously reported biodiesel from, Cannabis sativa methyl ester biodiesel^53,54^. It can be applied as an alternate as well as an eco-friendly fuel for diesel engines. Nevertheless, the very deepest characterization of ignition constraints for biodiesel blends will confidently give highlight the generosity of biodiesel that could be lastly castoff in I.C. engines in the years to bring and stunned the drawbacks of the gasoline diesel fuel that could be profitably developed.

**Table 2:** Comparison of the present study.

Emissions					Performances		
HC (ppm)		CO (%)	NO_x_ (ppm)	CO2 (%)	BTE (%)	BSFC (kg/kW h)	EGT ($^\circ{\rm C} )$
D10	45		50	4.8	28.2	0.8	500
D20	40	0.13	100	4.9	28.1	1.70	> 500
D30	44	0.14	150	4.8	27.9	2.60	> 500
D40	46	0.17	250	4.56	25.5	3.0	> 500
D100	41	0.18	350	4.9	24.1	3.4	> 500
BaseDiesel	43	0.21	–	6.1	23.6	3.5	550

## Conclusions

Throughout the investigation several tests were carried out, diesels engines can achieve acceptably with R. communis seeds biodiesel and their blend deprived of any engine adjustments. The oil yield attained from the R. communis seeds through the mechanical expeller was found to be 40%. Alkali base transesterification was performed for the manufacturing of biodiesel from R. communis oil. BTE for D10 and D20 tells almost similar to the base diesel. BSFC for D20 is the same as base diesel. Carbon-monoxide (CO) and hydrocarbon (HC) releases are higher for base diesel and lower for all the blends due to greater oxygen (O_2_) comfortable. Carbon dioxide(CO_2_) emissions for D10 and D20 were low compared to base diesel only at the lower loads situation were as for higher load Carbon dioxide(CO_2_) emissions were increasing. Oxides of nitrogen (NOx) emanations of all biodiesel blends are greater than of base diesel. The Oxides of nitrogen (NOx) releases of D10 were enlarged by 2% associated with diesel at the esteemed loads. Thus, results indicate that R. communis biodiesel could be a promising substitute and eco-friendly fuel for diesel engines. Though, characterizations of combustion parameters for biodiesel blend will confidently bounce importance on types of biodiesel that could be completely recycled in I.C. engine in the times in order to overwhelm the drawbacks of the firewood diesel fuels that profitably industrialized as well.

## Data Availability

The datasets used and analysed during the current study available from the corresponding author on reasonable request.
